# ELANE: an emerging lane to selective anticancer therapy

**DOI:** 10.1038/s41392-021-00766-2

**Published:** 2021-10-01

**Authors:** Boqiang Peng, Jiankun Hu, Xianghui Fu

**Affiliations:** 1 Laboratory of Gastric Cancer and Department of Gastrointestinal Surgery, State Key Laboratory of Biotherapy and Cancer Center, West China Hospital, Sichuan University and Collaborative Innovation Center of Biotherapy, Chengdu, Sichuan China; 2 Division of Endocrinology and Metabolism, State Key Laboratory of Biotherapy and Cancer Center, West China Hospital, Sichuan University and Collaborative Innovation Center of Biotherapy, Chengdu, Sichuan China

**Keywords:** Cancer, Immunotherapy

Recently, Cui et al.^1^ revealed that neutrophil elastase (ELANE) can selectively kill a wide range of cancer cells while sparing proximal non-cancer cells and significantly attenuate tumorigenesis, suggesting a promising selective and broad anticancer strategy.

Broad-spectrum anticancer therapies are challenging due to the spatial and temporal tumor heterogeneity. Innate immunity, with broad efficacy and specificity, holds great promise for developing broad anticancer therapeutics. Neutrophils, also known as polymorphonuclear cells (PMNs), the most prevalent immune cells that act as crucial effectors of innate immunity, are of particular interest due to their ability to eliminate genetically diverse pathogens.

Cui et al. showed that human PMN media under serum-free conditions (apoptotic PMNs) can kill 35 different human or murine cancer cells across 11 tumor types but spare healthy cells (Fig. 1). Combination of a quantitative killing assay and shotgun proteomic analysis, the authors then identified ELANE, a serine protease expressed in neutrophil azurophil granules, as the major protein mediating this anticancer activity. Intriguingly, ELANE can not only selectively induce cell death in diverse cancer cells, but not in normal cells, through liberating the CD95 death domain (DD) that interacts with histone H1 isoforms, but also prevent distant metastasis through triggering an abscopal effect mediated by CD8^+^ T cells. These new findings collectively provide an intriguing avenue for broad anticancer therapies and new insights into selective cancer cell killing.

**Fig. 1:**
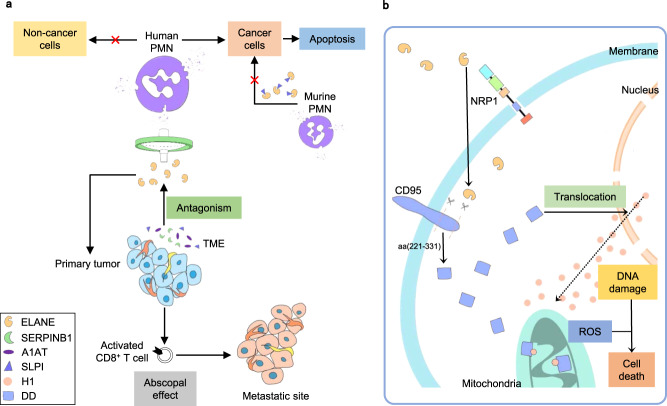
ELANE kills cancer cells without toxicity to non-cancer cells and induces cell apoptosis in primary tumors and at metastatic sites. **a** Human, but not murine, PMN media induces cancer cell death. In murine PMNs, co-released SLPI silences ELANE’s anticancer function. Similarly, certain serpins secreted by cells in the tumor microenvironment can antagonize ELANE’s cancer cell killing capability, such as SERPINB1, A1AT, and SLPI. ELANE can not only attenuate proximal tumorigenesis but also attack distal metastasis via activating CD8^+^ T cells. **b** The cellular uptake of ELANE by cancer cells depends on neuropilin-1 (NRP1). ELANE then induces apoptosis of cancer cells through the proteolytical liberation of the death domain (DD, aa: 221–331) of CD95 (DD^ELANE^). DD^ELANE^ induces DNA damage, which subsequently leads to the mitochondrial translocation histone H1. The formation of the H1-DD^ELANE^ complex may mediate reactive oxygen species at mitochondria and play a vital role in the anticancer selectivity of ELANE

Deserving attention is the cancer-selective property of ELANE. With great interest, Cui et al. provided several lines of evidence that may contribute to this selective toxicity. First, higher levels of CD95 could be observed in cancer cells. Second, expressions of histone H1 isoforms are generally increased in malignant cells compared with non-cancer cells. Third, there is an interaction between CD95-DD and histone H1 isoforms in cancer cells, but not in non-cancer cells. Taken together, these results strongly suggest that histone H1 isoforms play an essential role in ELANE’s selective toxicity against cancer cells. Furthermore, Cui et al. showed that ELANE can enhance the expression of γ-H2AX, a biomarker for DNA damage, which is able to trigger the mitochondrial translocation of histone H1. DNA damage endangers genome stability and predisposes to cancer, and its level is higher in malignant cells than in normal cells. Therefore, it is of interest in the future to clarify whether γ-H2AX participants in the process of ELANE-mediated H1 translocation.

Curiously, Cui et al. found that apoptotic PMN media from diverse human sources, but not that from murine sources, can inhibit tumor growth. Mechanistically, secretory leukocyte peptidase inhibitor (SLPI), a serpin (serine protease inhibitor), is co-released with ELANE in murine neutrophils, but not in human neutrophils, and SLPI can bind to ELANE, thereby resulting in the loss of ELANE activity in murine neutrophils. The species specificity and mechanistic insights throw new light on conceptualizing the contentious roles of neutrophils in tumor development.^2^ It also provides a reasonable explanation for the oncogenic function of ELANE observed in *ELANE* knockout mice.^3^ In addition, it is interesting to note that the timing of ELANE treatment may influence its functional consequences. Longer time points (6–24 h) of ELANE treatment induces cancer cell death, while the shorter time point (1 h) promotes cellular proliferation.^3^ Therefore, it is of importance to explore the optimized timing of ELANE treatment with the aim of its clinical application as an anticancer treatment option.

In this study, the combination of diverse mouse models clearly demonstrated that ELANE can attenuate tumor growth in vivo. However, this in vivo therapeutic efficacy of ELANE is much attenuated compared with its remarkable in vitro potency, raising the possibility that SLPI or other serpins may inactivate extracellular ELANE activity in vivo. Indeed, this study revealed that three serpins secreted by cells in the tumor microenvironment (TME), including A1AT, SERPINB1, and SLPI, can antagonize ELANE’s anti-tumor capability in a dose-dependent manner. In addition, most of circulating ELANE could be neutralized by the serum that contains some endogenous serpins. Hence, the presence of serpins in serum and TME may represent one of the potential obstacles of ELANE therapy. In line with this, porcine pancreatic elastase, an ELANE homolog showing less susceptibility to serpins, exhibited remarkably improved therapeutic efficacy in vivo, exemplifying a promising direction to optimize ELANE therapy. Of note, the crosstalk between ELANE and serpins has been implicated in many physiological and pathological processes. For instance, the imbalance of ELANE and serpins might lead to inflammatory lung diseases.^4^ In this regard, it is of importance to evaluate the potential toxicity of ELANE to non-cancer cells in vivo in long-term ELANE-based therapy.

Metastasis accounts for most of the cancer-related death. Interestingly, this study showed that ELANE therapy on the primary tumor can attack distant metastases, which is not due to ELANE spillover. This so-called abscopal effect is vanishingly rare, which is ascribed to the established immune-tolerance mechanisms.^5^ Therefore, it is highly commendable to identify the abscopal effect of ELANE. Combined with observations of ELANE’s non-toxicity to immune cells and previous findings that ELANE uptake by cancer cells can result in enhanced activation of cytotoxic CD8^+^ T cells, Cui et al. demonstrated that ELANE attenuates distant tumorigenesis via activating cytotoxic CD8^+^ T cells, which is independent of immune reaction. Although much remains to be furtherly explored, the innate immune system initiated by ELANE could drive adaptive immunity. Currently, the growing consensus is that a combination of immunotherapy with radiotherapy can boost the abscopal effect.^5^ Hence, ELANE therapy supplemented with radiotherapy perhaps holds significant potential to improve patient outcomes.

Despite these tremendous advances, this anticancer pathway of ELANE may require experimental validation in more cell types. Future studies aimed at the mechanism by which histone H1 and CD95-DD complexes trigger apoptosis will shed more light on the cancer-selective property of ELANE. Moreover, combination therapies with ELANE that could improve in vivo efficacy are worthy of further exploration.

In short, the study by Cui et al. demonstrates the great value and potential of ELANE therapy. Moreover, it provides new mechanistic insights into broad and selective anticancer therapy and paves the way for new therapeutic strategies.

## References

[1] Cui C (2021). Neutrophil elastase selectively kills cancer cells and attenuates tumorigenesis. Cell.

[2] Coffelt SB, Wellenstein MD, de Visser KE (2016). Neutrophils in cancer: neutral no more. Nat. Rev. Cancer.

[3] Houghton AM (2010). Neutrophil elastase-mediated degradation of IRS-1 accelerates lung tumor growth. Nat. Med..

[4] Genschmer KR (2019). Activated PMN exosomes: pathogenic entities causing matrix destruction and disease in the lung. Cell.

[5] Ngwa W (2018). Using immunotherapy to boost the abscopal effect. Nat. Rev. Cancer.

